# Molecular Response in Intestinal and Immune Tissues to *in Ovo* Administration of Inulin and the Combination of Inulin and *Lactobacillus lactis* Subsp. *cremoris*

**DOI:** 10.3389/fvets.2020.632476

**Published:** 2021-02-03

**Authors:** Aleksandra Dunislawska, Agnieszka Herosimczyk, Adam Lepczynski, Petr Slama, Anna Slawinska, Marek Bednarczyk, Maria Siwek

**Affiliations:** ^1^Department of Animal Biotechnology and Genetics, UTP University of Science and Technology, Bydgoszcz, Poland; ^2^Department of Physiology, Cytobiology, and Proteomics, West Pomeranian University of Technology, Szczecin, Poland; ^3^Department of Animal Morphology, Physiology and Genetics, Mendel University in Brno, Brno, Czechia

**Keywords:** microbiome, broiler chicken, gene expression, metabolism, immune response, inulin, inulin-based synbiotic

## Abstract

Intestinal microbiota are a key factor in maintaining good health and production results in chickens. They play an important role in the stimulation of immune responses, as well as in metabolic processes and nutrient digestion. Bioactive substances such as prebiotics, probiotics, or a combination of the two (synbiotic) can effectively stimulate intestinal microbiota and therefore replace antibiotic growth promoters. Intestinal microbiota might be stimulated at the early stage of embryo development *in ovo*. The aim of the study was to analyze the expression of genes related to energy metabolism and immune response after the administration of inulin and a synbiotic, in which lactic acid bacteria were combined with inulin in the intestines and immune tissues of chicken broilers. The experiment was performed on male broiler chickens. Eggs were incubated for 21 days in a commercial hatchery. On day 12 of egg incubation, inulin as a prebiotic and inulin with *Lactobacillus lactis* subsp. *cremoris* as a synbiotic were delivered to the egg chamber. The control group was injected with physiological saline. On day 35 post-hatching, birds from each group were randomly selected and sacrificed. Tissues (spleen, cecal tonsils, and large intestine) were collected and intended for RNA isolation. The gene panel (*ABCG8, HNF4A, ACOX2, APBB1IP, BRSK2, APOA1*, and *IRS2*) was selected based on the microarray dataset and biological functions of genes related to the energy metabolism and immune responses. Isolated RNA was analyzed using the RT-qPCR method, and the relative gene expression was calculated. In our experiment, distinct effects of prebiotics and synbiotics following *in ovo* delivery were manifested in all analyzed tissues, with the lowest number of genes with altered expression shown in the large intestines of broilers. The results demonstrated that prebiotics or synbiotics provide a potent stimulation of gene expression in the spleen and cecal tonsils of broiler chickens. The overall number of gene expression levels and the magnitude of their changes in the spleen and cecal tonsils were higher in the group of synbiotic chickens compared to the prebiotic group.

## Introduction

Bioactive substances such as prebiotics and probiotics are gaining increasing recognition as an alternative to antibiotic growth promoters. Vaccines and antibiotics are the commonly preferred methods of disease prevention and control in poultry. However, there are risks associated with the acquisition of antibiotic resistance by microorganisms through the excessive use of antibiotics. Current trends in poultry production focus on natural feed additives that would have a positive impact not only on production but also on animal health.

Bacterial microbiota in the avian digestive tract are a key factor in the development and regulation of immunity, in the digestion and absorption of nutrients, and in their metabolism (1, 2). Under breeding conditions, the first contact of the chicken digestive system with exogenous bacteria occurs after hatching. It is possible to change the composition of the gut microbiota, provided that this process is carried out early enough, and this influences changes in the metabolism later in the development of the organism (3). *In ovo* technology enables the administration of bioactive substances during embryonic development and the stimulation of the intestinal microbiota before hatching (4).

Inulin is one of the most commonly used prebiotic additives with widely reported effectiveness. The mechanism of its action within the host organism is multidirectional. However, the positive effect of inulin administered both traditionally as a feed additive (5) and *in ovo* at the stage of embryonic development (6) has been repeatedly demonstrated. The administration of inulin affects the modulation of the intestinal microbiota by promoting beneficial *Lactobacillus* and *Bifidobacterium* bacteria strains, and also by inhibiting the development of pathogenic microorganisms (7, 8).

Development of the chicken immune system begins during embryogenesis and is continued after the post-hatch period. The development of immunity requires not only antigen stimulation but also energy to maintain the immune organs' growth (9). There has been speculation that, in avian species, the size and weight of the spleen reflect immune maturity (10). The activity and development of the immune system cells are strongly correlated with cholesterol flux (11). On the other hand, the spleen is one of the organs involved in cholesterol metabolism. The importance of the spleen in cholesterol homeostasis is indicated by the results of studies showing changes in plasma cholesterol concentration in case of splenectomy and splenomegaly. Spleen resection causes hyperlipidemia, while, in contrast, one of the consequences of splenomegaly is hypolipidemia (12). The most common explanation for hypolipidemia in splenomegaly was the comparison of the spleen to a storage pool for lipids (12). Moreover, cholesterol and its metabolites are important factors in shaping immune response (13). Cecal tonsils are considered to be the largest lymphoid aggregates of the lymphoid tissue associated with the intestines in birds. They elicit an immune response against bacterial and viral pathogens in the chicken gastrointestinal tract (14). The main function of the large intestine is fermentation of indigestible food matter by bacteria. The metabolic functions of each section of the gastrointestinal tract are determined by the community of small organisms living there.

The aim of this study was to analyze the expression of genes related to energy metabolism and immune response after the administration of inulin and a synbiotic, in which lactic acid bacteria were combined with inulin in the intestines and immune tissues of chicken broilers. The novelty of these studies is a multitissue and long-term analysis of changes in the expression of metabolic and immunological genes in response to the administration of inulin on day 12 of egg incubation (*in ovo* stimulation).

## Materials and Methods

### *In ovo* Injection of Prebiotics and Synbiotics

The experiment was performed on 75 male broiler chickens (Ross 308, Aviangen Inc., Huntsville, AL, USA) as described in Slawinska et al. (15). Eggs were incubated for 21 days in a commercial hatchery. On day 12 of egg incubation, *in ovo* injection to an air chamber with 200 μL of bioactive substances was performed. Two bioactive substances were injected: prebiotic (PRE)—inulin extracted from *Dahlia tubers* (Sigma-Aldrich, GmbH, Schnelldorf, Germany); and synbiotic (SYN)—composed of 1.76 mg/egg of inulin and 1,000 CFU/egg *Lactobacillus lactis* subsp. *lactis* IBB2955 (IBB, PAS, Warsaw, Poland). Eggs were randomly selected to two experimental groups. The control group was injected with physiological saline. Bioactive substances preparation is described in Slawinska et al. (15). After hatching, the sex of each chick was determined. Thirty males with a mean initial body weight of 42 g were selected from each group for further steps. The selected birds were raised in three separate pens (one treatment group per pen) in accordance with the producer's standard rearing protocol for 35 days on a commercial farm. Straw was used as litter. Birds in all three groups underwent a three-phase feeding program. A starter ration was fed on days 1–14, a grower ration was fed on days 15–30, and a finisher ration was fed on days 31–35. Feed and water were provided *ad libitum*. Animal handling methodologies were approved by the Local Ethical Committee for Animal Experimentation, UTP University of Science and Technology in Bydgoszcz, Poland (Permit No 22/2012, June 21, 2012), and these were in accordance with the animal welfare recommendations of the European Union (directive 86/609/EEC).

### Sample Collection and RNA Extraction

On days 1, 14, and 35 post-hatching, birds from each group (n=5) were randomly selected and sacrificed. Tissues (spleen, cecal tonsils, and large intestine) were collected from each individual and fixed in liquid nitrogen, and then stored at −80°C for analysis. Frozen tissues were homogenized in a Trizol reagent (Invitrogen, Carlsbad, USA) using a TissueRuptor homogenizer (Qiagen GmbH, Hilden, Germany) for RNA extraction. Total RNA was purified with a Universal RNA Purification Kit (EURx, Gdansk, Poland) according to the manufacturer's instructions. Qualitative and quantitative control was performed using agarose gel electrophoresis and a NanoDrop 2,000 spectrophotometer (Thermo Scientific NanoDrop Products, Wilmington, USA). RNA samples were stored at −20°C.

### Gene Selection and Primer Design

The gene panel was selected based on the microarray dataset (Chicken Gene 1.1 ST, Affymetrix, Santa Clara, CA, USA) (15) and biological functions of genes related to the energy metabolism and immune responses. The analysis was carried out based on gene lists generated by Affymetrix Expression Console software and published in Slawinska et al. (15). *In silico* selection of gene sequences was based on the following criteria: *p*-value (*p* < 0.05) and fold change (up- or downregulated genes with a specific function related to metabolism and immune response after substance treatment). Sequences of primers were based on the literature or were designed with an NCBI Primer-BLAST tool based on the NCBI sequence. Primer sequences are shown in Table 1.

**Table 1:** Primer sequences used in the RT-qPCR reaction.

**Gene**	**Gene ID**	**Primer sequence**	**References**
*UB*	396190	F: GGGATGCAGATCTTCGTGAAA R: CTTGCCAGCAAAGATCAACCTT	(16)
*ACTB*	396526	F: CACAGATCATGTTTGAGACCTT R: CATCACAATACCAGTGGTACG	(17)
*G6PDH*	428188	F: CGGGAACCAAATGCACTTCGT R: GGCTGCCGTAGAGGTATGGGA	(17)
*ABCG8*	421402	F: TGCTCTGGAACCCAGGAATA R: CGGGGCTGATGAAGTGAAAG	This study
*HNF4A*	419198	F: TGCTGGGAGGTTCATCAAGT R: GCATCTGAGGAGGCATTGTG	This study
*ACOX2*	416068	F: AGACATGGGAAGGTCAGCAA R: TCCTGCAGTTATACCTGGGC	This study
*APBB1IP*	420492	F: CCTTATCAGCAGGAGCTGTTC R: AATGACCCGGGGAATCTGTC	This study
*BRSK2*	423098	F: TGAAGTTGGGGGTTCACTGT R: CGCTCCACCTTCATTAGCAC	This study
*APOA1*	396536	F: GGCAAACAGCTTGACCTGAA R: CCTCCTTGTAGTAGGGAGCC	This study
*IRS2*	101749060	F: CAGCCAAGGCCTTTTTCCAAG R: TGCCACTGACATACGCTATCC	This study

### RT-qPCR Reaction

Isolated RNA was reverse-transcribed to cDNA (Thermo Scientific, Maxima First Strand cDNA Synthesis Kit for RT-qPCR; Thermo Scientific/Fermentas, Vilnius, Lithuania) and diluted. Mixtures for qPCR contained the following: Maxima SYBR Green qPCR Master Mix (Thermo Scientific/Fermentas, Vilnius, Lithuania), 140 ng of cDNA, 1 μM of forward primer, and 1 μM of reverse primer. Primer sequences were derived from literature data or designed based on a cDNA nucleotide sequence using NCBI Primer Blast (18). qPCRs were carried out in duplicate. Thermal programs were carried out in a LightCycler II 480 (Roche Diagnostics, Basel, Switzerland). The program consisted of initial denaturation (95°C for 20 min) followed by 40 cycles of amplification (15 s at 95°C), annealing (20 s at melting temperature for each pair of primers), and elongation (20 s at 72°C). At the end of the thermal cycling, melting curves were generated to test for specificity of reactions.

### Relative Quantification of Gene Expression

Relative gene expression analysis was performed for each experimental group using the ddCt method (19) according to the formula R = 2^−ddCt^ using *UB, ACTB*, and *G6PDH* as reference genes. Geometric means of cycle threshold (Ct) values of reference genes were used in the analysis. Ct differences between target and reference genes were calculated for each sample. Control samples (injected with saline) were used as calibrators. The significance of the gene expression data was determined by comparing the Ct value of each experimental group with the control by Student's *t*-test (*P* < 0.05).

## Results

**Figure 1:**
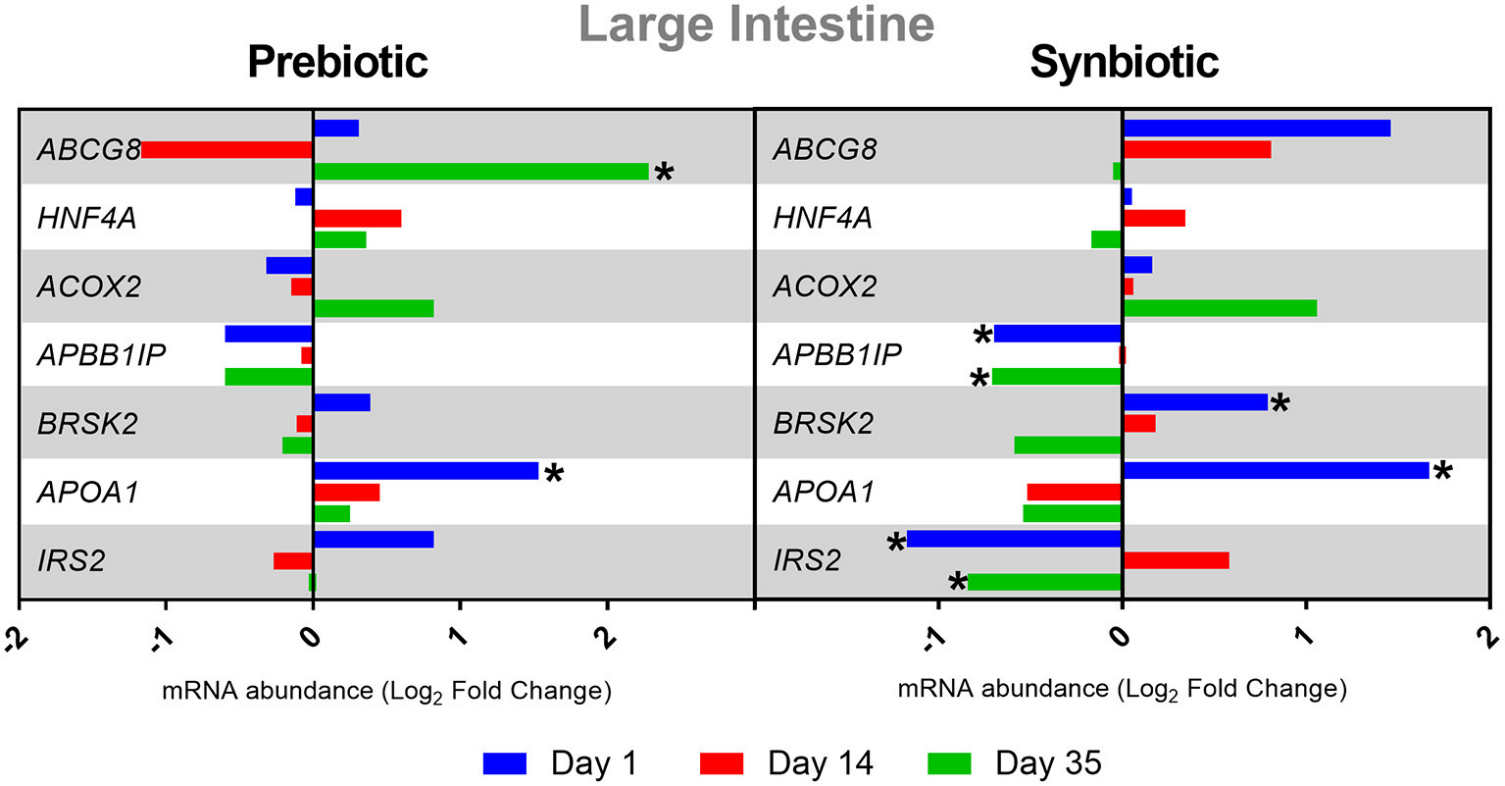
Relative mRNA expression in three time points (day 1, day 14, and day 35) of genes related to energy metabolism and immune response in large intestine of broiler chicken injected *in ovo* with prebiotic (inulin) and synbiotic (inulin with *Lactobacillus lactis* subsp. *lactis*).

The administration of the prebiotic *in ovo* caused a statistically significant increase in the expression of *ABCG8* on day 35 and *APOA1* on day 1 in the large intestine (*P* < 0.05). *In ovo* delivery of the synbiotic resulted in a negative regulation of *APBB1IP* and *IRS2* expression on days 1 and 35, and a positive regulation of *BRSK2* and *APOA1* on day 1 in the large intestine (*P* < 0.05). The results of gene expression in the large intestine are presented in Figure 1.

**Figure 2:**
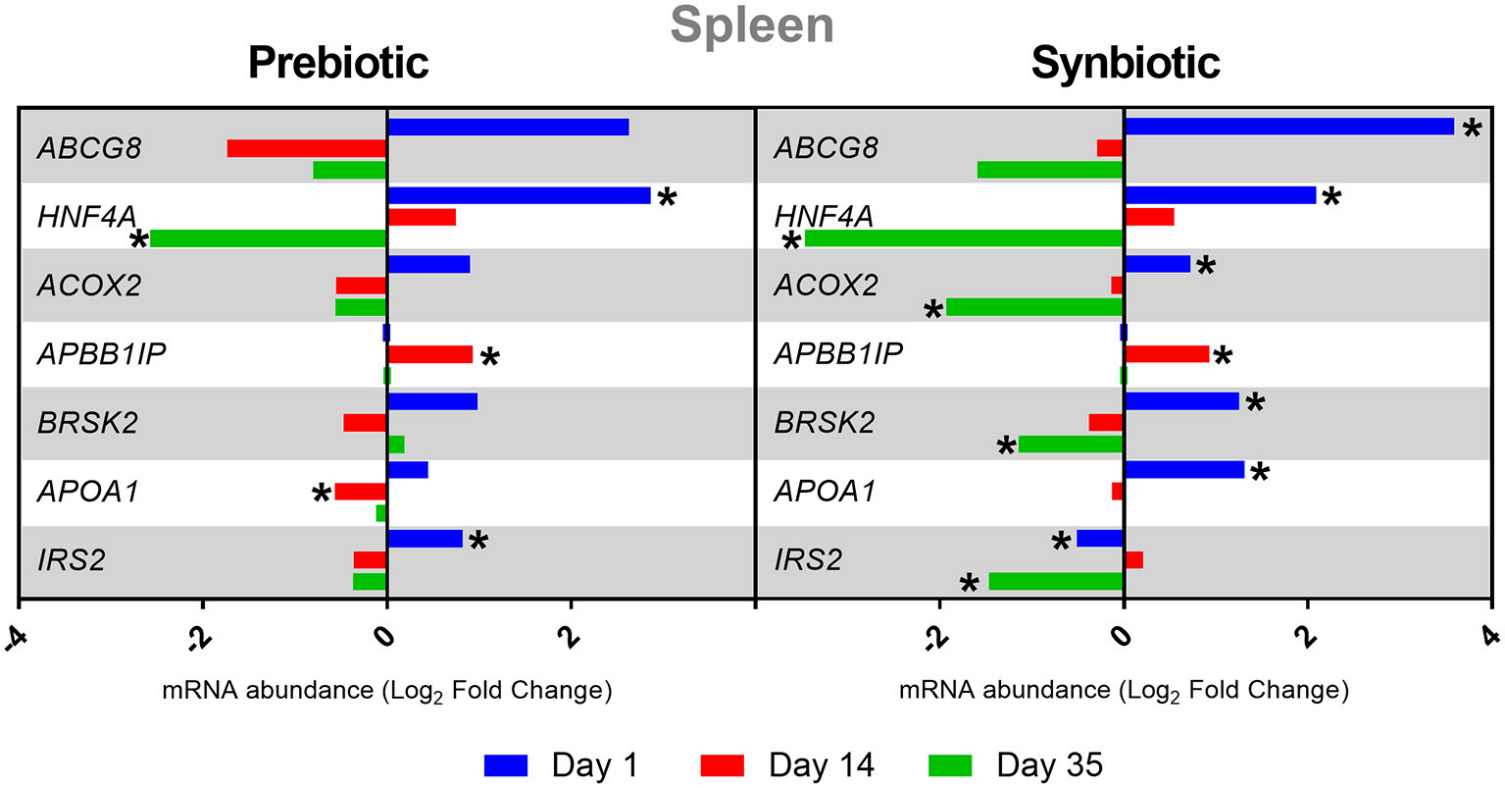
Relative mRNA expression in three time points (day 1, day 14, and day 35) of genes related to energy metabolism and immune response in spleen of broiler chicken injected *in ovo* with prebiotic (inulin) and synbiotic (inulin with *Lactobacillus lactis* subsp. *lactis*).

After the administration of the prebiotic, the spleen showed downregulation of the *HNF4A* gene on day 35 and of *APOA1* on day 14, and upregulation of *HNF4A* and *IRS2* on day 1 and of *APBB1IP* on day 14 (*P* < 0.05). The administration of the synbiotic showed increased expression of the following genes in the spleen on day 1: *ABCG8, HNF4A, ACOX2, BRSK2*, and *APOA1*. In contrast, there was a decrease in the expression of the *IRS2* gene (*P* < 0.05). On day 14, the *APBB1IP* gene was upregulated (*P* < 0.05). However, on day 35, the following genes showed downregulation: *HNF4A, ACOX2, BRSK2*, and *IRS2* (*P* < 0.05). The results of the changes in gene expression levels at three time points in the spleen are presented in Figure 2.

**Figure 3:**
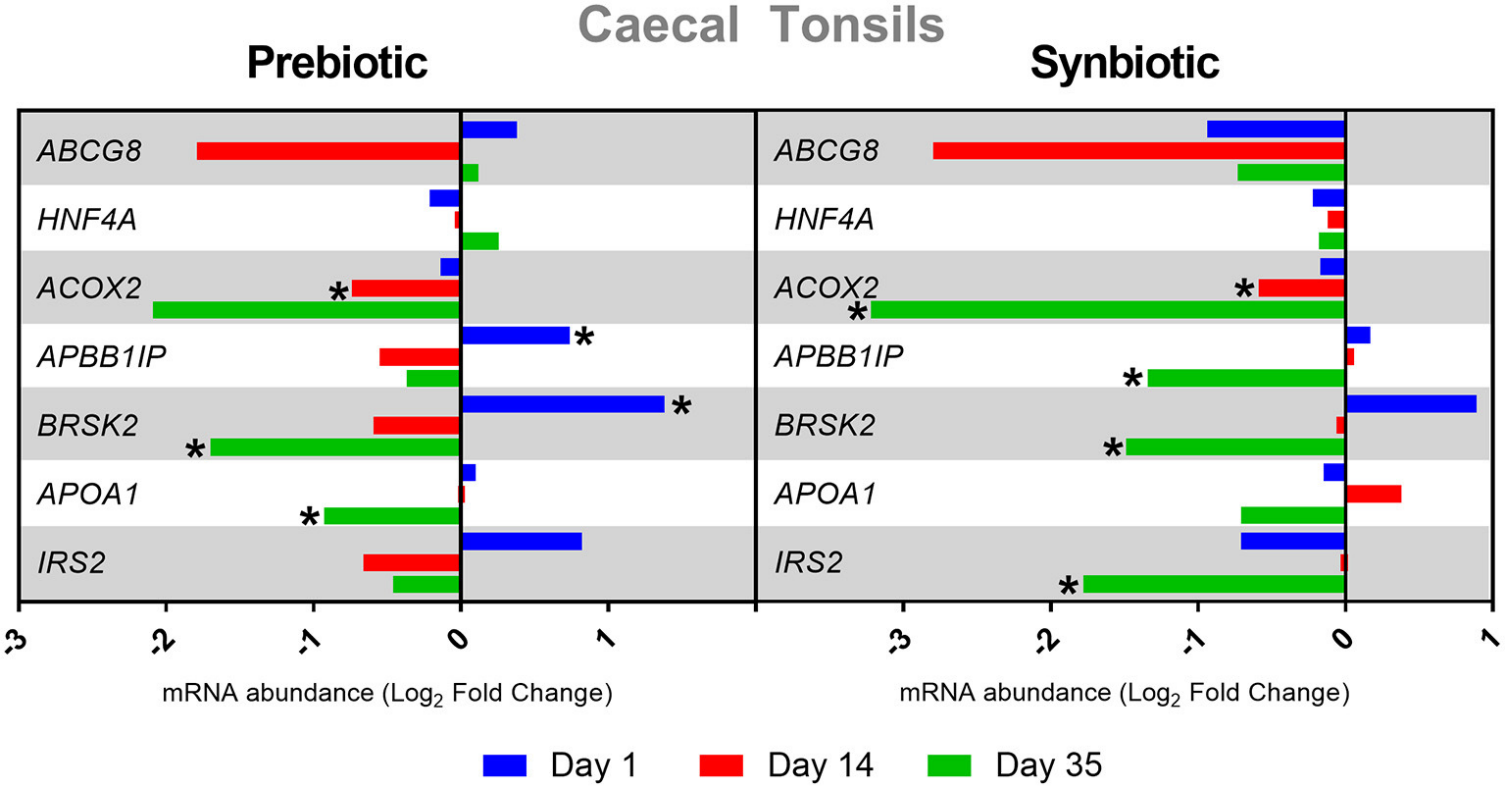
Relative mRNA expression in three time points (day 1, day 14, and day 35) of genes related to energy metabolism and immune response in cecal tonsils of broiler chicken injected *in ovo* with prebiotic (inulin) and synbiotic (inulin with *Lactobacillus lactis* subsp. *lactis*).

After administration of the prebiotic in cecal tonsils, negative expression of the *APOX2* gene was determined on day 14 and of the *BRSK2* and *APOA1* genes on day 35, while positive expression of *APBB1IP* and *BRSK2* was noted on day 35 (*P* < 0.05). After administration of the synbiotic, *ACOX2* expression was negatively regulated on days 14 and 35, and *APBB1IP, BRSK2*, and *IRS2* expression on day 35 (*P* < 0.05). The results of changes in gene expression in cecal tonsils after *in ovo* administration of substances are presented in Figure 3.

## Discussion

*In ovo* stimulation with microbiota-promoting bioactive substances is being rapidly adopted as the method of choice for modulating gut health, thereby enhancing immune system maturation in poultry (6, 20). Such early intervention is crucial for optimizing long-term health and performance effects as it allows for a proper transition in the early life stages from the embryonic to the early post-hatching periods of birds (21). Based on our previous reports, this technology proved to be efficient in delivering various bioactive substances including inulin prebiotics and inulin-based synbiotics into the developing chicken embryo (15, 22, 23). Moreover, these data demonstrated that a single *in ovo* delivery of prebiotics or synbiotics to a chicken embryo on day 12 of egg incubation significantly affected the central and peripheral lymphatic organ morphology and development (22, 23), and triggered transcriptional responses in the gut and immune-related tissues of adult individuals (15). Therefore, in this study, based on our previous transcriptome data (15), seven candidate genes related to energy metabolism and immune response were selected for further validation using RT-qPCR analysis. Results of the current study clearly indicate that the molecular responses through the modulation of gene expression are highly dependent on the type of bioactive substances used for *in ovo* stimulation, and this effect is stronger for inulin combined with *Lactobacillus lactis* (SYN) compared to inulin (PRE) administered alone. This can be attributed to the different modes of action displayed by prebiotics and synbiotics (24). Synbiotics are a combination of prebiotics and probiotics that are known to exert a synergistic interaction that likely leads to increased growth of indigenous beneficial gut microbiota as well as improved survival of probiotic microorganisms (6). Therefore, *in ovo* injection of synbiotics more effectively stimulates the host microbiome, which indirectly influences changes in gene expression in various tissues of broiler chickens. This is in accordance with our previous studies (15, 23), where superior effects of *in ovo* stimulation with synbiotics on the gene expression profile in the gut and immune-related tissues were found compared to their respective prebiotics.

### Intestinal Gene Expression Changes in Response to *in ovo* Stimulation With PRE or SYN

In the current experiment, distinct effects of PRE and SYN following *in ovo* delivery were manifested in all analyzed tissues, with the lowest number of genes with altered expression shown in the large intestines of broilers. More than half of the examined genes in the large gut did not show a consistent direction of their expression changes in the prebiotic and synbiotic groups at different time points after hatching. Nevertheless, *in ovo* stimulation with inulin was demonstrated to increase the expression of genes involved in cholesterol metabolism and transport such as *ABCG8* and *APOA1*. This could be attributed to the substantial metabolic changes occurring in the embryo in the hatching transition period in chickens (25). One of the underlying mechanisms involved in this process is a synergistic remodeling of a key hepatic mitochondrial network in order to sustain an undistorted switch from free fatty acids in the chick embryo to lipogenesis in the neonate (26). A concomitant increase in the mRNA levels for genes engaged in lipogenesis including cholesterol is also observed in the liver of newly hatched chicks (27). In the current study, *in ovo* treatment with both PRE and SYN induced an increased expression of the intestinal *APOA1* gene as early as day 1, and this was followed by a gradual decrease until the end of this experiment. This gene encodes apolipoprotein A-1 (apo A-I), a protein that, in chickens, is the main protein constituent of the high-density lipoprotein (HDL) and that, to a lesser extent, is also present in the very low-density lipoprotein (VLDL), intermediate density lipoprotein (IDL), and low-density lipoprotein (LDL) (28). Increased apo A-I expression has been shown to efficiently promote reverse cholesterol transport from various peripheral tissues and from the yolk sac *via* plasma HDL to the liver (29). It should also be emphasized that apart from the liver, the intestines are also an important site of apo A-I synthesis and secretion in chickens (28). Recent research has also revealed that intestine-derived apo A-I is transported via endosomes and lysosomes to the liver where it is accumulated, and this process has been confirmed to work bidirectionally (30). Changes in *APOA1* gene expression observed in the current study may be associated with digestive and metabolic adaptations as a rapid transition from yolk lipid-based metabolism to a solid carbohydrate-rich grain-based metabolism occurs over the first 2 weeks post-hatching (26). The period of short-term starvation before placement with feed and water might be another possible explanation for the observed *APOA1* gene expression changes. This is in accordance with a recent proteomic investigation by Simon et al. (31) where 24-h-long fasting was shown to cause an upregulation of Apo A-I in the jejunum of broiler chicken. Additionally, there are data indicating that both intestinal microbiota as well as gut microbiome-derived short-chain fatty acids (SCFAs) are also a potent stimulus of intestinal and liver lipoprotein formation (32, 33). Taken together, the dynamic changes of *APOA1* gene expression demonstrated in our study could be attributed to a concomitant combination of the aforementioned mechanism.

Our further analysis revealed increased *ABCG8* gene expression in the large intestine of 35-day-old broiler chickens injected *in ovo* with inulin. The ATP-binding cassette (ABC) genes represent the largest family of transmembrane proteins that are key components involved in lipid homeostasis as they are implicated in the ATP-dependent transport of cholesterol, bile acids, phospholipids, and sphingolipids (34). A study by Yu et al. (35) demonstrated that intestinal cholesterol absorption was profoundly reduced, whereas its biliary secretion and fecal excretion were shown to be increased in transgenic mice overexpressing the human ABCG8 protein. Therefore, we believe that the significant upregulation of the *ABCG8* gene observed in the current study provides another premise supporting the idea that prebiotics exert a hypocholesterolemic effect in broiler chickens.

Furthermore, our data also identified two genes (*IRB2* and *APBB1IP*) that displayed a higher level of expression in the group of 1- and 35-day-old chickens that received *in ovo* SYN. The *IRB2* gene encodes insulin receptor substrate 2, an adaptor protein that is known to play an important role in glucose homeostasis via insulin signal transduction (36). It had previously been shown that some species of *Lactobacilli* had the ability to attenuate, *inter alia*, type 2 diabetes through increasing the mRNA level of *IRS2* in rodents (25, 37). This is also in accordance with our previous study where *in ovo* injection with synbiotics, i.e., *Lactobacillus salivarius* with galactooligosaccharides (GOS) or *Lactobacillus plantarum* with raffinose family oligosaccharides (RFO), caused an improved plasma glucose homeostasis and insulin sensitivity in 7-day-old broiler chickens (38). Although *IRS2* is known primarily for its insulin-induced metabolic effects, there are results confirming its additional role in the processes of intestinal epithelial cell proliferation and differentiation (39, 40). An immunohistochemical study by Modica et al. (39) showed that the *IRS2* protein is predominantly expressed in the ileal villus and at the surface of the colonic epithelium of adult mice. Moreover, these authors also revealed highly elevated expression of *IRS2* mRNA and protein levels in HT29 cells after 24-h treatment with sodium butyrate at 1 mM concentrations (39). A more recent study by Anders et al. (40) shed new light on the role of the IRS2 protein for proper gut function as they demonstrated that high *IRS2* expression is crucial for maintaining a regenerative capacity of the small intestinal and colonic epithelium through homeostatic mechanisms that keep the balance between proliferative and mature cells. Additionally, Anders et al. (40) also postulate that decreased *IRS2* expression in the small intestinal or colonic epithelium may serve as an early potential marker for tumor development. Here, we also demonstrated that SYN delivered *in ovo* induced an increase in the expression of the *APBB1IP* gene encoding the Rap1-interacting molecule (RIAM). The RIAM protein plays an important adaptor role in the proper assembly of adhesion complexes as its depletion has been shown to attenuate the interactions between focal adhesion components (41). It is known that RIAM can be activated through phosphorylation by the focal adhesion kinase (FAK) (42). As reported by Ma et al. (43), elevated FAK activity is required in both the maintenance and repair of the epithelial barrier through the redistribution of tight junctional (TJ) proteins. As shown by Liu et al. (44), microbiota-targeted interventions such as prebiotics, probiotics, and synbiotics administration have been proven to regulate intestinal epithelial function by increasing TJ formation. Therefore, we assumed that the upregulation of both *IRB2* and *APBB1IP* genes could further confirm the above-mentioned findings indicating that synbiotics are key players in maintaining the regenerative capacity of the large intestinal epithelium as well as in keeping the intestinal barrier at steady state in broiler chickens.

### Gene Expression Changes in The Peripheral Immune Organs Triggered by *in ovo* Stimulation With PRE or SYN

Our results also demonstrated that PRE or SYN injected *in ovo* provides a potent stimulation of gene expression in the spleen and cecal tonsils of broiler chickens. The overall number of gene expression levels and the magnitude of their changes in the spleen and cecal tonsils were higher in the group of SYN chickens compared to the PRE group. However, unlike in the case of the large intestines, the direction of their expression changes was largely consistent in both analyzed tissues. According to the above, it seems that the observed trend of gene expression alterations in peripheral immune organs may reflect a dynamic and critical phase in shaping both the immune and metabolic system in the early post-hatching period.

Cytokines are critical intercellular mediators of communication in the immune system. They are also known to be the main regulators of the initiation and maintenance of the host immune defense and homeostasis (45). As shown by Sugimura et al. (46), *Lactococcus lactis* JCM5805 can stimulate human dendritic cells for the production of IFNs. This is consistent with our previous study where *in ovo* stimulation with inulin enriched with *Lactococcus lactis* 2955 caused a significant upregulation of the IFN-γ gene in the cecal tonsils but not in the spleen of 35-day-old chickens (23). Cytokine-induced immune response is also regulated by the hepatocyte nuclear factor 4 alpha (*HNF4A*) gene, as previously reported by Ihara et al. (47) and Jiang et al. (48). *HNF4A* is present in leukocytes, granulocytes, and T cells (48), which are the source of many cytokines that play an important role in the modulation of inflammatory response. In our experiment, the splenic *HNF4A* gene was shown to be upregulated in 1-day-old chickens and was followed by a significant down-expression at 35 days post-hatch in both experimental groups. This gradual decrease in *HNF4A* expression could be related to the fact that in 35-day-old chickens, the spleen is fully mature and allows for stable immune function maintenance. Our data also demonstrated that both PRE and SYN delivered *in ovo* caused an increased expression of a splenic *APBB1IP* gene encoding Rap1-interacting molecule (RIAM) in the group of 14-day-old chickens. This could be potentially associated with a dramatic change in the immune system of chickens that occurs at about 14 days of age. Chickens after hatching are well equipped with antibodies from their mothers, and their levels rise to a peak at around 14 days and subsequently fall with time to a level that is not sufficient to protect against various pathogens. This time is very stressful for the chicken body and stimulation for immune system development. The *APBB1IP* protein is a key activator of leukocyte integrins (49) and has also been shown to induce β1 and β2 integrin-mediated adhesion in Jurkat T cells (50). Additionally, Klapproth et al. (51) demonstrated an important role of *APBB1IP* in β2 integrin activation in neutrophils, macrophages, and T cells in mice.

Here, we also found *APOA1* gene expression changes in the spleen and cecal tonsils in both experimental groups. Apart from its role in lipoprotein metabolism, the apo A-I protein also exerts anti-inflammatory properties (52). In the spleen of 1-day-old chickens, *in ovo* stimulation with inulin or an inulin-based synbiotic induced an upregulation of the *APOA1* gene; however, the changes were confirmed to be statistically relevant only in the SYN group. The increased *APOAI* expression on the first day after hatching may be caused by the factors previously described in the case of the large intestine: adaptation from yolk lipid-based metabolism and simultaneous short post-hatch food deprivation that causes reverse cholesterol transport from the spleen and other immune tissue. The same mechanism may be proposed for *ABCG8* expression. On the other hand, it may constitute another evidence of the anti-inflammatory effects of synbiotics containing *Lactococcus lactis*. At 14 days after hatch, the direction of *APOA1* gene expression was changed toward downregulation in the group of birds treated *in ovo* with inulin. This suggests that prebiotics and, to some extent, synbiotics can weaken anti-inflammatory functions in favor of pro-inflammatory reactions, which can be associated with changes to the immune system of chickens with a positive effect on monocyte recruitment and macrophage chemotaxis. Decreased levels of *APOA1* gene expression were also observed in the cecal tonsils of 35-day-old chickens in both experimental groups; however, these changes did not reach statistical significance in the PRE group. This reflects that the immune system of chickens at that age is not only concentrated on the anti-inflammatory responses related to monocytes and macrophages. Specific immunity is much better developed. This finding is further supported by the decreased expression of *IRS2* that was found in spleen and cecal tonsils in the group of 1- and 35-day-old chickens stimulated *in ovo* with SYN. The IRS2 protein is mainly involved in IL-4-induced proliferation and has well-documented antiapoptotic properties (53, 54). This is consistent with our previous study where the relative expression of IL-4 in the cecal tonsils of 35-day-old chickens was also found to be downregulated in response to *in ovo* stimulation with inulin alone or inulin enriched with *Lactococcus lactis* 2955 (23). It is known that IL-4 is an anti-inflammatory cytokine, and its downregulation shows the orientation of the immune system to the specific responses too. Moreover, it was recently reported by Bohlul et al. that *Lactococcus lactis* can induce cellular apoptosis (55). Therefore, we believe that during the development of the immune system, aberrant immune cells can be eliminated by apoptosis, which may be modulated by *Lactococcus lactis* via *IRS2* downregulation.

Analyses carried out in the same experiment showed a positive effect of inulin on the length of intestinal villi, including increased activity of amylase, lipase, and trypsin in the pancreas, and improved short-chain fatty acid profile. It was also shown to increase the number of goblet cells in the duodenum and the jejunum (6, 56). Earlier reports on the *in ovo* administration of inulin suggested that it may modulate the development of the central and peripheral lymphatic organs in broilers alone or in combination with a probiotic. This injection also leads to the development of GALT after hatching and colonization of lymphocytes. In cecal tonsils, on day 7, stronger colonization of GALT by T lymphocytes was observed after the administration of the inulin and the inulin-based synbiotic. On day 21, an increase in the number of T lymphocytes in the ileum was also demonstrated. Inulin does not affect the quality characteristics of the meat. After administration of the inulin-based synbiotic, the growth rate of the chickens was faster (1–3 weeks) compared to the saline-treated control group (6, 57). In conclusion, the present study showed that *in ovo* treatment with an inulin-based synbiotic proved to be effective at modulating gene expression changes in the peripheral immune and gut tissues in broiler chickens. The synbiotic used in this experiment composed of inulin and *L. lactis* appeared to be potent in stimulating the interactions between microbiota and host cells. Our data also confirmed favorable effects of *in ovo* prebiotics and synbiotics stimulation on improved gut barrier integrity and lipid metabolism, as reflected by a significant upregulation of the *ABCG8, IRB2*, and *APBB1IP* genes in the large gut.

## Data Availability Statement

The original contributions generated in the study are included in the article/supplementary materials, further inquiries can be directed to the corresponding author.

## Ethics Statement

The animal study was reviewed and approved by Permit No. 22/2012, June 21st 2012.

## Author Contributions

AD performed molecular analysis (RNA isolation, quantitative and qualitative evaluation, designed primers for the reaction, and performed RT-qPCR reactions) and processed the results. AD, AH, AL, and PS wrote the main manuscript text. MS developed the concept of the manuscript, revised the main text of the manuscript, and approved the final version for publication. AS prepared all the figures. AS and MB revised the manuscript. MB made available biological material for analysis from his project. AD and MS obtained research funding. All authors contributed to the article and approved the submitted version.

## Conflict of Interest

The authors declare that the research was conducted in the absence of any commercial or financial relationships that could be construed as a potential conflict of interest.
